# Bridging the gap - planning Lifestyle Medicine fellowship curricula: A cross sectional study

**DOI:** 10.1186/s12909-014-0271-4

**Published:** 2014-12-30

**Authors:** Rani Polak, Marie L Dacey, Hillary Keenan, Edward M Phillips

**Affiliations:** Institute of Lifestyle Medicine, Physical Medicine and Rehabilitation, Harvard Medical School, Joslin Diabetes Center, One Joslin Place, Boston, MA USA; School of Arts & Sciences, MCPHS University, 179 Longwood Ave, Boston, MA USA; Joslin Diabetes Center, Harvard Medical School, One Joslin Place, Boston, MA USA

**Keywords:** Lifestyle medicine, Curriculum, Fellowship, Medical education

## Abstract

**Background:**

The emerging field, Lifestyle Medicine (LM), is the evidence-based practice of assisting individuals and families to adopt and sustain behaviors that can improve health. While competencies for LM education have been defined, and undergraduate curricula have been published, there are no published reports that address graduate level fellowship in LM. This paper describes the process of planning a LM fellowship curriculum at a major, academic teaching institution.

**Methods:**

In September 2012 Harvard Medical School Department of Physical Medicine and Rehabilitation approved a “Research Fellowship in Lifestyle Medicine”. A Likert scale questionnaire was created and disseminated to forty LM stakeholders worldwide, which measured perceived relative importance of six domains and eight educational experiences to include in a one-year LM fellowship. Statistical procedures included analysis of variance and the Wilcoxon signed-rank test.

**Results:**

Thirty-five stakeholders (87.5%) completed the survey. All domains except smoking cessation were graded at 4 or 5 by at least 85% of the respondents. After excluding smoking cessation, nutrition, physical activity, behavioral change techniques, stress resiliency, and personal health behaviors were rated as equally important components of a LM fellowship curriculum (average M = 4.69, SD = 0.15, p = 0.12). All educational experiences, with the exception of completing certification programs, research experience and fund raising, were graded at 4 or 5 by at least 82% of the responders. The remaining educational experiences, i.e. clinical practice, teaching physicians and medical students, teaching other health care providers, developing lifestyle interventions and developing health promotion programs were ranked as equally important in a LM fellowship program (average M = 4.23, SD = 0.11, p = 0.07).

**Conclusions:**

Lifestyle fellowship curricula components were defined based on LM stakeholders’ input. These domains and educational experiences represent the range of competencies previously noted as important in the practice of LM. As the foundation of an inaugural physician fellowship, they inform the educational objectives and future evaluation of this fellowship.

## Background

By 2020, the World Health Organization predicts that two-thirds of all disease worldwide will be the result of lifestyle choices [1]. In the United States, the primary causes of premature, adult deaths are related to lifestyle such as tobacco use (18.1%) and poor diet and lack of physical inactivity (15.2%) [2]. These findings are widely accepted, and well-established chronic disease practice guidelines uniformly call for lifestyle change as the first line of prevention and management [3,4]. However, physicians often do not follow these recommendations [5,6]. Barriers include lack of time, compensation, knowledge, and resources [7,8].

The emerging field, Lifestyle Medicine (LM), was defined in the Journal of American Medical Association (JAMA) as the “evidence-based practice of assisting individuals and their families to adopt and sustain behaviors that can improve health and quality of life” [9]. In addition, it states that although environmental and community factors have crucial roles in creating and sustaining appropriate health behaviors, it does not eliminate the duty of physicians to assist patients in making health behavior changes [9]. This position was supported in recent surveys with both patients [10] and physicians [11]. A model of care, similar to the Patient Centered Medical Homes [12], was suggested for delivering LM, in that a physician is a coordinator of an inter-professional health care team and does not operate solely on a one-to-one basis with patients [13].

One of the causes identified for the lack of practicing LM is the dearth of physician education in LM competencies, and increasing medical education was proposed as a solution. Goals for primary care LM education were defined accordingly and include competencies in leadership, knowledge, assessment skills, management skills and the use of office and community support. Although these suggested competencies were developed largely to guide Continuing Medical Education (CME) for primary care and preventive care physicians, physician educators at both the undergraduate and graduate medical education were encouraged to incorporate them into LM training programs [9]. LM curricula have started to be incorporated into medical schools [14-16] and CME programs [17] as well. These curricula have been described and empirically evaluated [17,18]. However, there are no published reports describing graduate level fellowships in LM although they have been suggested [19,20].

Fellowship programs in other medical fields, such as wound care and palliative medicine, have begun as initiatives within specific institutions [21], and sometimes as a research fellowship [22]. As programs within a field, such as in Geriatrics [23] and Emergency Medicine [24], became more widespread and evidence-based, experts from the relevant professional associations collaborated and defined fellowship training curricular components. This process of standardization has led to proposals and eventual accreditation by the Accreditation Council for Graduate Medical Education (ACGME) and the American Board of Medical Specialties (ABMS).

Wehrli [25] suggested stages in the process of developing curricula. The first three stages, “identifying the problem”, “performing needs assessment” and “defining goals and objectives” were already defined for LM education as described previously [9]. However, the fourth stage “developing the program contents”, has not yet been defined for LM fellowships. Other fellowship curricula have been developed after literature searches have indicated relevant content [26], or when institutions or organizations have stated necessary components [22].

This paper describes the process of planning a curriculum for a LM fellowship initiative at a major, academic teaching institution, based on stakeholders’ opinions regarding important components. This systematic approach to planning this fellowship will hopefully provide guidance for other LM training programs.

## Methods

To establish an appropriate syllabus for a one-year fellowship, the authors developed a pilot survey based on the LM competencies that were previously defined [9]. Because specific lifestyle domains (e.g., diet, exercise, smoking, etc.) were not included in the published competencies, the authors first assessed perceived importance of specific domains. The second part of the survey assessed perceived importance of the educational experiences needed in a one-year LM curriculum in order to achieve these competencies. The survey was pilot-tested with multidisciplinary professionals in the Institute of Lifestyle Medicine, Joslin Diabetes Center, Harvard Medical School [27] that included a nutritionist, psychologist, sports physician and family physician. The final version of the questionnaire included 14 five-point Likert scale items (1 - not important; 5 - very important); six items measured attitudes regarding suggested LM domains and eight items addressed recommended LM educational experiences. In addition, the survey participants were invited to add narrative comments to each section.

For stakeholder selection, the authors first identified five focus areas and three leadership areas to be represented. Focus areas were medical education (students and residents), research, health policy, primary care, and LM practice. Leadership areas were LM, professional societies (LM, preventive medicine and primary care), and medical education (faculty). A convenience sample of 40 stakeholders was identified for this pilot study to include at least 10% from each focus and leadership area and at least 20% from outside North America (Table 1).

**Table 1:** **Responders professional characteristics**

	**Responders n = 40**
Focus areas	
Medical education (trainee)	4 (10%)
Research	7 (18%)
Health policy	6 (15%)
Primary care	8 (20%)
LM practice	4 (10%)
Leadership areas	
Lifestyle Medicine	18 (45%)
Professional societies	15 (38%)
Medical education (faculty)	17 (43%)
Countries of activity	
North America	30 (75%)
Others	10 (25%)

All characteristics: n, (%).

In December 2013 the first author (RP) emailed an online survey link to these 40 stakeholders. Two follow-up emails were sent in January 2014. Data were collected and entered into an Excel spreadsheet (Excel 2010; Microsoft Corp., Seattle, Washington), which was then used to create a dataset for statistical analysis. The Statistical Analysis System v 9.2 (Cary, NC) was used for all analyses. This cross section analysis granted an exemption from requiring ethics approval by the Joslin Diabetes Center Committee on Human Studies.

**Figure 1:**
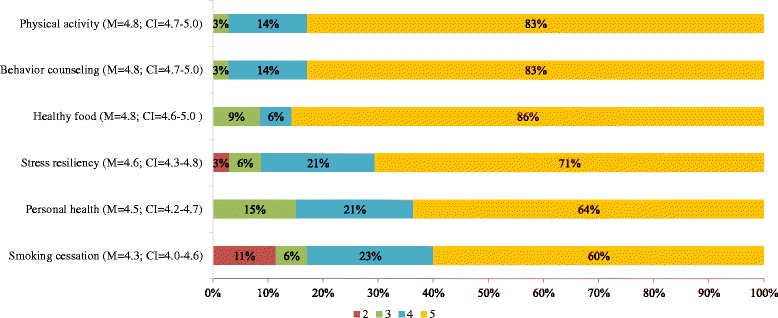
**Lifestyle medicine domains.** Percent of responders’ perceived importance of specific LM domains to include in LM fellowship curricula, (1 - not important; 5 - very important), M = mean, CI = 95% Confidence Interval.

**Figure 2:**
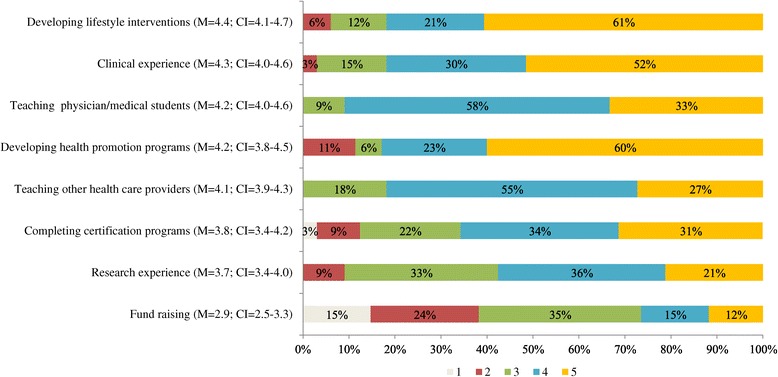
**Lifestyle medicine educational experiences.** Percent of responders’ perceived importance of specific LM educational experiences to include in LM fellowship curricula, (1 - not important; 5 - very important), M = mean, CI = 95% Confidence Interval.

**Figure 3:**
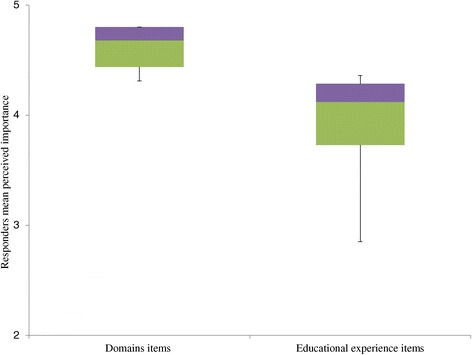
**Domains Vs. Educational experiences.** Responders’ perceived importance of LM domains compared to LM educational experiences (P < 0.01). Upper horizontal line of box, 75th percentile; lower horizontal line of box, 25th percentile; horizontal bar within box, median; upper horizontal bar outside box, maximum; lower horizontal bar outside box, minimum.

Within-item responses were analyzed categorically using a chi-square test for homogeneity (Figures 1 and 2). Equity in variance within each educational item (e.g. LM domains and educational experiences) was calculated using the analysis of variance (ANOVA) (Figures 1 and 2). Comparison of the attitudes between educational items was calculated using Wilcoxon signed-rank test for non-normally distributed data (Figure 3). P-values ≤0.05 were considered statistically significant.

## Results

Forty stakeholders were identified with professional characteristics presented in Table 1. Thirty-five (87.5%) of them completed the survey (a minimum of 32 responses for each item). The distributions of attitude and average mean regarding the importance of including specific domains in a LM fellowship curriculum are presented in Figure 1. All domains except smoking cessation, i.e. nutrition, physical activity, behavioral change techniques, stress resiliency and personal behavior, were graded at 4 or 5 from at least 85% of the responders. Analysis of variance shows significant difference overall among all the domains (p = 0.02). However, after excluding smoking cessation the difference is non-significant among the other 5 domains (M = 4.7, p = 0.12).

Attitudes distribution and average mean regarding the importance of including specific educational experiences in the fellowship curriculum are presented in Figure 2. Five educational experiences, i.e. clinical practice, teaching physicians and medical students, teaching other health care providers, developing lifestyle interventions, and developing health promotion programs, were graded at 4 or 5 from at least 82% of the responders. The remaining three were graded at 4 or 5 by less than 65%. Analysis of variance among the educational experiences show that there is significant difference among them (p < 0.001). However when excluding the three that received the lowest importance (completing certification programs, research experience, and fund raising) the difference among the remaining educational experiences does not reach statistical significance (M = 4.23, p = 0.07).

Comparison between the responders’ average perceived importance of the LM domains (median = 4.68; interquartile range = 0.36; range = 0.49) and the educational experiences (median = 4.12; interquartile range = 0.56; range = 1.51) is presented in Figure 3. It shows that the responders’ attitude regarding the importance of the LM domains was significantly higher than the educational experiences (p < 0.01) and that its distribution was smaller.

Twenty-four responders (68.6%) wrote narrative suggestions in the “other domain” field, eight (22.9%) in the “other educational experience” field and five (14.3%) wrote general comments. Major LM domain suggestions included practice care delivery models and reimbursement, sleep medicine and mindfulness. Specific behavior change techniques such as coaching, motivational interviewing, Cognitive Behavioral Therapy (CBT), empathy, culture of change, and group dynamics were mentioned as well. Major educational experience suggestions were practicing LM as part of a multi-disciplinary health care team and experiencing self-health behavior. The main general recommendation was the importance of using existing knowledge like the JAMA LM competencies [9] or existing programs such as Dean Ornish Program [28] or Coronary Health Improvement Program (CHIP) [29].

## Discussion

We assessed the perceived importance of LM domains and educational experiences to be included in a LM fellowship curriculum. Overall, it appears that the responders were positive about the domains and the educational experiences as well as about developing such a curriculum for implementation.

Reports describing the emerging field of LM [13,30] and the JAMA Competencies [9] do not define specific domains that constitute LM. However, the American College of Lifestyle Medicine (ACLM) specifies the domains of diet, exercise, stress management and smoking cessation as components of the field of LM [31]. In our survey results, two domain areas warrant particular attention.

The responders’ attitude regarding the importance of including smoking cessation as part of LM fellowship curriculum was significantly lower than other LM domains. This finding aligns with previous findings indicating that smoking cessation education is inconsistently included in LM curricula [15,17]. Results here and previously may reflect the perception that the gap in smoking cessation medical education is not as large as other LM domains as there has already been initiatives that build it into medical education [32,33]. Further research is needed to define whether smoking cessation should be included in LM training programs and if so to what extent and how to optimally integrate this into existing training opportunities on this topic.

The only LM domain in which responders suggested specific content was behavioral change techniques. While describing LM, Egger notes that nutrition and physical activity are the “penicillin” of LM, while behavioral change techniques are the “syringe” through which these are delivered [13]. We suggest that in addition to discussing which health behaviors should compose the “penicillin” (e.g. domains) in the emerging field of LM, a different discussion should occur about which are the best evidence based “syringes” (e.g. counseling techniques) to deliver those messages. Knowledge and skills development in behavioral counseling should be part of any future LM curriculum. Thus, we suggest that future efforts, including surveys, to further define LM fellowship curricula should address clarifying which specific health behaviors as well as behavioral change techniques to include.

The American College of Lifestyle Medicine (ACLM), suggested development of competencies based fellowship in LM that will train physician to become clinical specialist in LM [20]. However, in contrast to the more traditional solo practitioner model of care, the physician role in LM tends to rely on coordinating a team of certified health professionals [13] such as the evolving role of a physician within a Patient Centered Medical Home [12]. This might reflect the responders’ low importance scores regarding including specific certificates of completion as part of the physician medical education.

Mechanick et al. suggest that LM fellowship will train physicians to have “expertise”, which includes competency, knowledge, skills, and attitudes, but also a broader knowledge base, leadership roles, teaching, and original research [19]. With this perspective, LM expertise, in addition to clinical practice, will have educational components related to developing LM training programs and advocating for these programs at the undergraduate, graduate, and post-graduate levels [19]. This might explain the responders” high-perceived importance for program development and teaching experience, but does not explain the low importance of research education. Perhaps these ratings reflect the responders’ attitude that LM specialists should be positioned in the community as educators and clinicians.

Also, further evaluation needs to be done to understand why key gaps to LM implementation such as funding received low importance scores, especially when reimbursement was the most frequent domain mentioned in the narrative comments. Together with the variety of narrative suggestions for behavioral change techniques perhaps this reflects the tension between the desire to provide best practice, satisfying medicine and the need to be reimbursed.

Although twenty-four responders (68.6%) wrote narrative suggestions in the “Other domain” field, only eight responders (22.9%) wrote narrative suggestions in the “other educational experience” field. None of the popular innovative educational modalities such as “hands-on” labs [34] or simulated patients [35,36] were suggested, perhaps due to the design of the survey, which was predominantly with closed questions.

The responders’ average attitudes regarding the importance of the educational experiences was lower than their average domain attitude scores. This might reflect the relative infancy of the field of LM where the destination (e.g., healthy nutrition and adequate physical activity) is clear but the path of how to change and sustain the health behavior is less clear [19,20]. The lower average scores regarding the importance of the educational experience might be a reflection of different roles that different responders envision for a LM specialist/fellow graduate within the LM world and the whole medical arena.

There are limitations in the current study. First, this was a pilot study using a small convenience sample of multidisciplinary professionals. As a result, generalizability is limited, as we do not know to what degree the results reflected this particular participant pool. Second, as our focus was to identify the most important components for future training, we established cut points in terms of resource allocation. However, future studies should strive to use power calculation analysis to collect and assess whether grouping similar grading and isolating deviant responses is valid. The pilot data collected by this study could be used to obtain an estimate of effect needed to do a power calculation for future studies. Third, data collection optimally would have included breakdown by stakeholders’ leadership and focus areas in order to determine different training programs for different roles of the LM expert. Finally, although our sample includes a variety of disciplines and countries of origin, it is predominantly North American based, and it does not include patients’ perspectives.

Since this is an inaugural fellowship, a valid qualitative evaluation of the program cannot be conducted. However, each quarter the curriculum is being reevaluated based on the CanMEDS portfolio [37]. This record of participation and achievements in the fields of research, education and teaching, clinical experience, and career development and leadership will be used to evaluate the program. In addition, the results of this pilot study have informed the educational objectives of the fellowship as well, and thus will also contribute to the evaluation process.

## Conclusion

This article outlines our needs assessments while creating a one-year formal physician fellowship in Lifestyle Medicine. This curriculum’s domains have been defined according to the results and include nutrition, physical activity, stress resiliency, behavioral change and the fellow’s personal health while excluding smoking cessation. Its educational experiences have been focused on teaching physicians, medical students and other health care providers, clinical experience and developing lifestyle interventions and health promotion programs. Additional research has also been conducted and certificates have been completed, however these activities were only conducted as they were needed for program development or clinical purposes.

While this initial survey was conducted to contribute to the development of the program for the inaugural fellow, forming well-grounded fellowships creates benefits beyond those afforded to this individual physician. A draft curriculum has been outlined and will be published at the end of the LM fellowship. We hope that this work will be a cornerstone that will encourage other institutions to establish a LM fellowship program and that appropriate funding opportunities might follow. We believe that after further standardization, a LM fellowship program might be recognized by the ACGME toward accreditation by the ABMS as a subspecialty.

### Study design

Cross sectional study.

## Abbreviations

LM: Lifestyle medicine
JAMA: Journal of American Medical Association
CME: Continuing medical education
ACGME: Accreditation council for graduate medical education
ABMS: American board of medical specialties
ILM: Institute of lifestyle medicine
HMS-PM&R: Department of physical medicine & rehabilitation at Harvard medical school
ACLM: American College of lifestyle medicine
